# A Prospective Study of the Incidence of Myocarditis/Pericarditis and New Onset Cardiac Symptoms following Smallpox and Influenza Vaccination

**DOI:** 10.1371/journal.pone.0118283

**Published:** 2015-03-20

**Authors:** Renata J. M. Engler, Michael R. Nelson, Limone C. Collins Jr., Christina Spooner, Brian A. Hemann, Barnett T. Gibbs, J. Edwin Atwood, Robin S. Howard, Audrey S. Chang, Daniel L. Cruser, Daniel G. Gates, Marina N. Vernalis, Marguerite S. Lengkeek, Bruce M. McClenathan, Allan S. Jaffe, Leslie T. Cooper, Steve Black, Christopher Carlson, Christopher Wilson, Robert L. Davis

**Affiliations:** 1 Military Vaccine Agency-Vaccine Healthcare Centers Network (currently Defense Health Agency, Immunization Healthcare Branch), Walter Reed National Military Medical Center, Bethesda, Maryland, United States of America; 2 Department of Medicine and Pediatrics, Uniformed Services University of the Health Sciences, Bethesda, Maryland, United States of America; 3 Allergy-Immunology-Immunizations, Walter Reed National Military Medical Center, Bethesda, Maryland, United States of America; 4 Cardiology Service, Department of Medicine, Walter Reed National Military Medical Center, Bethesda, Maryland, United States of America; 5 Department of Medicine, Uniformed Services University of the Health Sciences, Bethesda, Maryland, United States of America; 6 Department of Research Programs, Walter Reed National Military Medical Center, Bethesda, Maryland, United States of America; 7 Department of Pathology, Vassar Brothers Medical Center, Poughkeepsie, New York, United States of America; 8 Cardiology Service, Fort Belvoir Community Hospital, Fort Belvoir, Virginia, United States of America; 9 Integrated Cardiac Health Project, Walter Reed National Military Medical Center, Bethesda, Maryland, United States of America; 10 Allergy and Asthma Care Centers, Chantilly, Virginia, United States of America; 11 Military Vaccine Agency-Vaccine Healthcare Centers Network (currently Defense Health Agency, Immunization Healthcare Branch), Womack Army Medical Center, Fort Bragg, North Carolina, United States of America; 12 Division of Cardiovascular Diseases, Mayo Clinic, Rochester, Minnesota, United States of America; 13 Cincinnati Children's Hospital Center for Global Health, Cincinnati, Ohio, United States of America; 14 Division of Public Health Sciences, Fred Hutchinson Cancer Research Center, Seattle, Washington, United States of America; 15 Department of Immunology, University of Washington, Seattle, Washington, United States of America; 16 Center for Biomedical Informatics, University of Tennessee Health Sciences Center, Memphis, Tennessee, United States of America; 17 Oak Ridge National Laboratory, Oak Ridge, Tennessee, United States of America; University of British Columbia, CANADA

## Abstract

**Background:**

Although myocarditis/pericarditis (MP) has been identified as an adverse event following smallpox vaccine (SPX), the prospective incidence of this reaction and new onset cardiac symptoms, including possible subclinical injury, has not been prospectively defined.

**Purpose:**

The study’s primary objective was to determine the prospective incidence of new onset cardiac symptoms, clinical and possible subclinical MP in temporal association with immunization.

**Methods:**

New onset cardiac symptoms, clinical MP and cardiac specific troponin T (cTnT) elevations following SPX (above individual baseline values) were measured in a multi-center prospective, active surveillance cohort study of healthy subjects receiving either smallpox vaccine or trivalent influenza vaccine (TIV).

**Results:**

New onset chest pain, dyspnea, and/or palpitations occurred in 10.6% of SPX-vaccinees and 2.6% of TIV-vaccinees within 30 days of immunization (relative risk (RR) 4.0, 95% CI: 1.7-9.3). Among the 1081 SPX-vaccinees with complete follow-up, 4 Caucasian males were diagnosed with probable myocarditis and 1 female with suspected pericarditis. This indicates a post-SPX incidence rate more than 200-times higher than the pre-SPX background population surveillance rate of myocarditis/pericarditis (RR 214, 95% CI 65-558). Additionally, 31 SPX-vaccinees without specific cardiac symptoms were found to have over 2-fold increases in cTnT (>99th percentile) from baseline (pre-SPX) during the window of risk for clinical myocarditis/pericarditis and meeting a proposed case definition for possible subclinical myocarditis. This rate is 60-times higher than the incidence rate of overt clinical cases. No clinical or possible subclinical myocarditis cases were identified in the TIV-vaccinated group.

**Conclusions:**

Passive surveillance significantly underestimates the true incidence of myocarditis/pericarditis after smallpox immunization. Evidence of subclinical transient cardiac muscle injury post-vaccinia immunization is a finding that requires further study to include long-term outcomes surveillance. Active safety surveillance is needed to identify adverse events that are not well understood or previously recognized.

## Introduction

More than 2 million U.S. service members have been immunized since December 2002 to prevent smallpox infection [1]. Over 290 million doses of smallpox vaccine (SPX) are available for immunization of the U.S. population in the event smallpox is used as an agent of bioterrorism [2]. Myocarditis and/or pericarditis (MP) have been causally linked to smallpox vaccination in retrospective epidemiological studies and clinical trials that led to FDA approval of the current cell culture derived vaccine [3–5]. Prelicensure study data for the newer cell culture derived vaccine suggested higher incidence rates of myocarditis/pericarditis (MP) following both the calf-lymph and cell culture derived vaccine product (ACAM2000) [5]. Based on a healthy cohort of service members with no prior vaccine exposure (primary vaccinees) in 2002–2003, the incidence rate of clinically diagnosed MP was estimated at 16.1 cases per 100,000 smallpox vaccine recipients, nearly 7.5-fold higher than the expected background rate of 2.16 per 100,000 among comparable unvaccinated service members [3]. An expanded Department of Defense (DoD) population-based estimate for clinically diagnosed MP in over 730,000 SPX vaccinees was approximately 12 per 100,000 [4]. Public health passive surveillance for adverse events following immunization may underestimate the true incidence of causally associated events [6, 7].

The primary objectives of this study were to determine the incidence of new onset cardiac symptoms, clinical myocarditis/pericarditis and cardiac specific troponin T (cTnT) increases following SPX immunization. Secondary objectives included comparison of the results to a pilot study of 200 subjects receiving annual trivalent influenza vaccine (TIV) and comparison of published cohort data related to background MP incidence rates in healthy service members not exposed to SPX [3].

## Methods

A multi-center, prospective, observational cohort study included healthy subjects receiving either the live attenuated smallpox (vaccinia) or an annual inactivated trivalent influenza (TIV) vaccine.

### Cohorts

Study participants were recruited by research nurses from healthy service members and Military Health System beneficiaries with occupational and/or clinical requirements for the vaccine at participating immunization clinics at study sites. Recruitment methods included local printed media advertisements as well as brief announcements about the study predominantly in immunization clinic waiting areas. All subjects were enrolled from August 2004 through June 2010 at multiple sites with written informed consent authorized by an institution-specific protocol. Approvals for this study were obtained from Institutional Review Boards at Walter Reed Army Medical Center, Washington, DC, Womack Army Medical Center, Fort Bragg, NC, and Brooke Army Medical Center, San Antonio, TX. We estimate about 5 to 20 thousand people were exposed to outreach and advertisement efforts. Telephonic and/or electronic mail follow-up to determine possible occurrence of new cardiac events following completion of study visits and blood draws (within 30 days following immunization) was conducted (starting between day 45–60) and was finalized by January 2011.

### Inclusion and Exclusion Criteria

Healthy active subjects presenting for either smallpox or annual influenza immunization as part of their military readiness and/or preventive health care requirements were considered for enrollment. Exclusion criteria included prior history of cardiac disease, diabetes, uncontrolled hypertension or specific medical exclusion for immunization. Military Health System standard of care guidelines for smallpox vaccine medical exemptions included a history of atopic dermatitis, cardiac disease or three or more cardiac risk factors, immune deficiency or immunosuppressive therapies, or any chronic illness potentially increasing the risk of vaccinia complications as assessed by clinical screening [8]. Both cohorts were without systemic symptoms of illness at the time of immunization and had no history of chest pains in the previous 72 hours. Additional exclusion criteria included pregnancy and age less than 18 years of age.

### Cohort Clinical Visits

Both study cohorts were evaluated during a pre-immunization visit and up to 2 post-vaccine visits (day 5–8 and/or day 9–28). Baseline data including age, race/ethnicity, sex, cardiac risk factors, atopic/medical history, and fitness assessments as measured by physical training abilities, were collected on the day of the SPX or TIV immunization. Clinical data including cardiac symptoms with visual analogue scale rating (0–10) of severity and 12-lead electrocardiograms (ECG) were collected at baseline and at up to two post-vaccine visits between days 5 and 30. Blood samples were collected by standardized venipuncture and rapidly processed (<1 hour) for specific testing. Sera were rapidly aliquoted and frozen to minus 80 degrees Celsius.

### Electrocardiograms

Standard 12-lead ECGs were performed using certified local clinic-based equipment. ECG tracings from all available visits were reviewed in a single session to rectify inaccurate machine reads when appropriate, and to validate changes from baseline. Potentially pathologic ECG changes were prospectively defined according to published criteria and included the following: (1) ST-segment elevation ≥1 mm (0.1 mV) elevation in two or more contiguous leads and not consistent with early repolarization (based on blinded consensus expert cardiology review), (2) T-wave changes (inversion, becoming negative) and*/*or (3) new arrhythmias (paroxysmal or sustained atrial or ventricular arrhythmias), (4) AV nodal conduction delays or intraventricular conduction defects, or (5) continuous ECG monitoring that detects frequent atrial or ventricular ectopy [9]. ECGs showing changes consistent with pericarditis and/or myocarditis underwent a second blinded review by a different cardiologist, along with a 4:1 sample of normal ECGs from the study subjects initially read as normal and of comparable age and sex. There were no disagreements noted between the 1^st^ and 2^nd^ readings performed in accordance with recommendations for standardization and interpretation of electrocardiograms published by the American Heart Association [9].

### Cardiac Biomarkers—cTnT

Samples for cTnT were measured using the Cobas Troponin T Short Turn-Around Time Cardiac T electrochemiluminescence immunoassay in a College of American Pathologists accredited laboratory. Monoclonal antibodies used in this system have minimal cross reactivity with non-cardiac troponin-T (h-skeletal muscle troponin T 0.001%, h-skeletal muscle tropomyosin 0.001%). The 99^th^ percentile cut-off for a normal level of cTnT (lower limit of detection) for this assay was <0.01 nanograms/milliliter (ng/ml) [10–13]. This cut-off level has been previously validated in normal populations with a coefficient of variation of <10%. The prevalence of cTnT levels ≥0.01 ng/ml published by Wallace et al. [12] for a civilian general population cohort (n = 3557) was 0.7%. Even subjects with minimal increases (0.01 to 0.02 ng/ml, compared to ≥0.03 ng/ml) had increased prevalence of cardiac comorbidities. It is noteworthy that a subsequent study with a high sensitivity cTnT assay (De Lemos [14, 15]) showed no difference in cTnT elevations between smokers and non-smokers, a potential confounder in younger service members. Serial samples from each subject were tested at the same time in the same assay run (thawed for the first time) to reduce inter-assay variability and to optimize for each subject the post vaccine comparison with pre-immunization cTnT levels as their personalized normal baseline control value.

### MP Diagnosis

Clinical myocarditis and pericarditis cases arising from the two prospective cohorts were independently adjudicated based on published epidemiological case definitions [16] that require the development of new onset cardiac symptoms in temporal association with vaccine exposure. Tables 1 and 2 outline the criteria for case definitions used in this study.

**Table 1:** Myocarditis case definition for surveillance of adverse events after smallpox vaccination in the United States, 2003^13^.

Evidence Level of Diagnostic Certainty	Signs & Symptoms	Testing	Imaging Studies^‡^	Histopathology
**Suspected Myocarditis** *Symptom onset within 4–30 days post smallpox vaccine (applies to all diagnostic levels of certainty)*	Dyspnea, palpitations, and/or chest pain of probable cardiac origin, in the absence of any other likely cause of symptoms	**Cardiac enzymes**: Normal or not performed* **ECG findings:** New, beyond normal variant^†^	Evidence of diffuse or focal depressed left ventricular function of indeterminate age	Not performed or normal
**Probable Myocarditis**	Dyspnea, palpitations, and/or chest pain of probable cardiac origin, in the absence of any other likely cause of symptoms	**Cardiac enzymes:** Elevated cTnT, cTnI or CK-MB* **ECG findings**: New, beyond normal variant^†^	Evidence of focal or depressed left ventricular function that is documented new onset or increased severity^‡^; myocardial inflammation	Not performed or normal
**Confirmed Myocarditis**	Dyspnea, palpitations, and/or chest pain of probable cardiac origin, in the absence of any other likely cause of symptoms	**Cardiac enzymes**: Not performed, normal or elevated* **ECG findings**: Not performed, normal or abnormal^†^	Not performed, normal, or abnormal	Evidence of myocardial inflammatory infiltrate with necrosis and myocyte damage

*Cardiac enzymes: Cardiac-specific troponin I (cTnI) or T (cTnT) preferred but includes creatine kinase-myocardial band (CK-MB).

^†^ **ECG findings**: Electrocardiogram findings (beyond normal variants) not previously documented to include ST-segment or T-wave abnormalities; paroxysmal or sustained atrial or ventricular arrhythmias; atrial ventricular nodal conduction delays or intraventricular conduction defects; continuous ambulatory electrocardiographic monitoring that detects frequent atrial or ventricular ectopy.

^‡^ **Imaging studies**: Include echocardiograms and radionuclide ventriculography using cardiac MRI with gadolinium or gallium-67; in absence of a previous study, findings of depressed left ventricular function are considered of new onset if, on follow-up studies, these findings improve or worsen.

**Table 2:** Pericarditis case definition for surveillance of adverse events after smallpox vaccination in the United States, 2003^13^.

Evidence Level of Diagnostic Certainty	Signs & Symptoms	New ECG Findings*	Echocardiogram	Histopathology
**Suspected Pericarditis** *Symptom onset within 4–30 days post smallpox vaccine (applies to all diagnostic levels)*	Typical chest pain (i.e., pain made worse by lying down and relieved by sitting up and/or leaning forward) in the absence of evidence of any other likely cause	Not performed, normal, or with preexisting or new abnormalities not described below*	Not performed, normal, or abnormalities not described below	Not performed or normal
**Probable Pericarditis**	Typical chest pain (i.e., pain made worse by lying down & relieved by sitting up &/or leaning forward) in the absence of evidence of any other likely cause; pleuritic or other chest pain not characteristic of any other disease; or pericardial rub	Diffuse ST-segment elevations or PR depressions without reciprocal ST depressions	Presence of an abnormal collection of pericardial fluid (e.g., anterior & posterior effusion or a large posterior effusion alone	Not performed or normal
**Confirmed Pericarditis**	Typical chest pain (i.e., pain made worse by lying down & relieved by sitting up &/or leaning forward) in the absence of evidence of any other likely cause; pleuritic or other chest pain not characteristic of any other disease; or pericardial rub	Not performed, normal or abnormal*	Not performed, normal, or abnormal	Evidence of pericardial inflammation

*ECG findings: Electrocardiogram findings not previously documented.

### Possible Subclinical Myocarditis/Pericarditis Case Definitions

Possible subclinical pericarditis was defined as new-onset characteristic ECG changes at any post-vaccine visit, in the absence of new-onset cardiac symptoms. Cardiac troponin T is a laboratory measure specific for myocardial injury with elevations above 0.01 ng/ml (10 ng/L) [10–13]. A single elevated cTnT measurement in a study of a general population has been associated with increased risk of mortality and cardiac morbidity on long-term follow-up [12]. New elevations were used as a surrogate biomarker of possible subclinical myocarditis in the prospective study populations. We defined possible subclinical myocarditis as the development of any one of the following: a) elevated post-vaccine levels of cTnT ≥ 0.02 ng/ml with pre-vaccine cTnT levels <0.01 ng/ml; or b) a post-vaccine cTnT level of 0.02 ng/ml greater than the pre-vaccine level based on the imprecision profiles of the assay. This conservative change value, double the lower limit of assay detection (0.01 ng/ml), was adopted to account for the imprecision profile of the cTnT assay equipment used to make the measurements.

### Comparison to Cohorts from Peer-Reviewed Studies

A 2002 background incidence of MP among 1,390,352 service members (no exposure to vaccinia) was used to calculate the relative risk of post-SPX clinical MP identified in this prospective study [3]. Table 3 provides further details about this reference control population (non-vaccinated) and data concerning the incidence rate of post-primary SPX MP in 347,516 SPX vaccinees between December 2002-September 2003. Cases in this SPX published cohort were identified over any 30-day post-vaccination period using ICD-9-CM (International Classification of Diseases, Ninth Revision) codes specific for the diagnoses of interest (420.90, 420.99, unspecified and other acute pericarditis; 422.90, 422.91, acute unspecified and idiopathic myocarditis; and 429.0, unspecified acute myocarditis).

**Table 3:** Characteristics of two (2) published cohorts of healthy adults providing data regarding the incidence rates of myocarditis/pericarditis (MP) per 100,000 (prior to or following SPX)^3^.

Characteristic	Healthy-2002 *	SPX-Vaccinees Primary ^†^
**Population Cohorts (Healthy)**	1,390,352	347,516
Active duty service members	Yes	Yes
Age, years: Mean (SD or Range)	27.8	29.3 (SD 8.4)
Time Frame of Enrollment	Dec 1, 2001-Nov 30, 2002	Dec 15, 2002- Sep 20, 2003
**SPX Immunization**	No	Yes
Percent primary vaccines	—	100%
**MP Cases**	30	*56*
Probable/confirmed case definition	**—**	56
*Relative Risk*, *unadjusted*		16.11
Rate per 100,000 (95% CI)	2.16 (1.9, 2.34)	7.46 (6.89, 8.48)

*Healthy 2002^3^: Uniformed service members whose medical encounters were recorded in the DoD Defense Medical Surveillance System pre-SPX immunization^3^; included both inpatient and outpatient cases diagnosed in military health system.

^†^ **SPX-Vaccinees^3^**: Uniformed service members within the DoD who received a primary (1^st^ time) SPX immunization.

### Statistical Analysis

For univariate analysis comparing cohorts, continuous and ordinal data were analyzed using the two-sample t-test. Categorical outcomes were examined using Fisher's exact test. Relative risks are presented with 95% confidence intervals (95% CI). The association of type of vaccine with new onset cardiac symptoms in the SPX and TIV cohorts, controlling for differences in baseline characteristics between groups, was examined using a log-binomial regression model. For analyses of clinical and subclinical MP, incidence rates and the 95% CI per 100,000 vaccinees (SPX and TIV cohorts) as well as relative risks (with 95% confidence intervals) were computed based on the Poisson distribution; cohorts were compared using the homogeneity test for Poisson rates. Data were analyzed from 2011 to 2013 using SPSS for Windows (v. 19 SPSS/IBM, Chicago IL) and StatXact (v.8 Cytel Inc., Cambridge MA).

## Results

**Fig 1:**
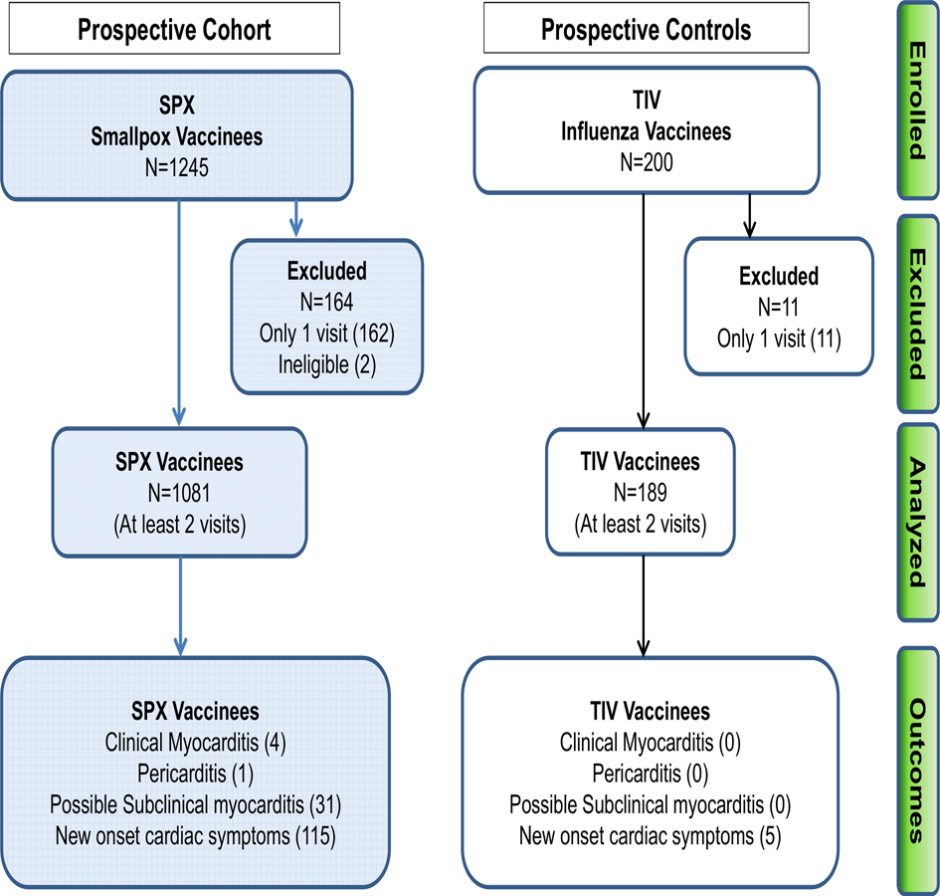
Subject enrollment, exclusions and outcomes for two prospective cohorts, post-smallpox and annual trivalent influenza vaccine.

Of 1245 volunteers enrolled in the SPX cohort and 200 in the pilot TIV cohort, 1081 SPX and 189 TIV vaccinees completed at least 2 visits (Fig. 1) and were used for analysis of incidence of new onset cardiac symptoms and clinical MP as well as dynamic changes in cTnT levels. In the SPX cohort, 164 were lost to follow-up after visit 1 due to scheduling conflicts and/or deployment. No subjects from the SPX and TIV cohorts presented acutely with clinical signs of MP prior to day 5 post-vaccine. Data was recorded on subject outcomes for at least 30 days following immunization by telephone or electronic mail following the last visit to assure that no cardiac symptoms or events occurred within the 30 day period after immunization.

Baseline assessments for the SPX and TIV cohorts are listed in Table 4. Subjects in the SPX cohort were younger, predominantly male and current or recent smokers. There was no significant difference in pre-immunization health self-assessment between groups. During the study period, there was no active participation in triathlons or prolonged endurance training exceeding the usual ongoing fitness training. Male subjects in the TIV cohort were heavier and older. The TIV cohort also self-assessed more frequently for physical limitations that presumably might reduce physical activity. However, all subjects in the TIV cohort were healthy, active adults, capable of meeting the physical fitness levels required for active duty service including completion of semi-annual physical fitness tests (adjusted for age and some physical limitations such as doing a fast walk rather than a run). The proportion of individuals with pre-vaccine baseline cTnT levels of ≥0.02 ng/ml (>2-fold higher than the >99^th^ percentile cut-off for the clinical assay) was not significantly different between TIV (0%) and SPX (1.0%) groups and comparable to the background rate in a healthy population [12, 14].

**Table 4:** Baseline demographic and clinical characteristics of smallpox (SPX) and trivalent inactivated influenza vaccine (TIV) cohorts including subjects with at least 2 visits (pre/post immunization).

Characteristic	SPX Vaccine	TIV Vaccine	P value:
	*n* = 1081	*n* = 189	*SPX-TIV*
**Sex *n (%)***			
Male	956 (88.4%)	102 (54.0%)	<0.001
Female	125 (11.6%)	87 (46.0%)	
**Age (years)**			
Mean (SD)	23.4 (5.7)	36.4 (11.3)	<0.001
**Race *n (%)***			
White	740 (68.5%)	117 (61.9%)	0.08
All Other Races	341 (31.5%)	72 (38.1%)	
**Vaccine Dose: Primary**	1027 (95.0%)	NA	
**Weight (lbs)** *			
Mean (SD)	175.7 (26.7)	173.4 (33.1)	0.37
**Smoking history *n (%)***			
Current/recent	581 (53.7%)	27 (14.3%)	<0.001
**Vaccine Type *n (%)***			
Dryvax	676 (62.5%)	NA	
ACAM2000	405 (37.5%)	NA	
Pre-Vaccine Health Self-Assessment*			
Excellent	637 (59.0%)	100 (52.9%)	0.23
Good	411 (38.0%)	85 (45.0%)	
Fair	32 (3.0%)	4 (2.1%)	
Poor	0 (0.0%)	0 (0.0%)	
**Physical Limitations**	77 (7.1%)	28 (14.8%)	<0.001

SD: Standard Deviation; NA: not applicable;

*missing data for one SPX subject.

### New Onset Cardiac Symptoms following Smallpox or Influenza Vaccine

The incidence of new onset cardiac symptoms following immunization with SPX and TIV is shown in Table 5. Despite no significant differences in pre-vaccine health self-assessment between the cohorts and fewer reported physical limitations in the SPX cohort, there was a significantly higher incidence of new onset cardiac symptom(s) post-SPX (10.6%) compared to post-TIV (2.6%), p<0.001. In univariate analysis, current/recent smoking status, presence of physical limitations and a lower self-reported health assessment were significantly associated with the onset of new cardiac symptoms post-vaccination (detailed in Table 6). Controlling for group differences in age, sex, weight, race, smoking, and physical limitations, the risk of having one or more new onset cardiac symptoms was greater following SPX than after TIV (adjusted relative risk (adjRR) 4.9, 95% CI: 1.9–12.8). This difference persisted when only new onset cardiac symptoms with severity of 3 or greater on the 10 point visual analogue scale were evaluated after adjusting for baseline variables (adjRR 5.4, 95% CI: 1.6–18.1). It is noteworthy that the most commonly reported new onset (possible) cardiac associated symptoms post-SPX are chest pain and dyspnea on exertion.

**Table 5:** Frequency of new onset cardiac symptoms day 4–30 after immunization with smallpox (SPX) versus trivalent inactivated influenza vaccines (TIV).

Characteristic	SPX	TIV	Relative Risk	P value:
	n = 1081	n = 189	(95% CI)	SPX-TIV
**New Onset Cardiac Symptoms**				
Chest Pain	87 (8.0%)	3 (1.6%)	5.1 (1.7–15.9)	<0.001
Dyspnea on Exertion	43 (4.0%)	0 (0.0%)		0.002
Dyspnea at Rest	13 (1.2%)	0 (0.0%)		0.24
Palpitations	12 (1.1%)	2 (1.1%)	1.0 (0.3–6.7)	0.99
**Any New Cardiac Symptoms**	115 (10.6%)	5 (2.6%)	4.0 (1.7–9.3)	<0.001
**Severe Cardiac Symptom(s)** *	95 (8.8%)	3 (1.6%)	5.5 (1.9–17.5)	<0.001

*Visual Analogue Scale severity >3/10 with symptom duration for at least 2 days.

**Table 6:** Characteristics of subjects with and without new onset cardiac symptoms.

Characteristic	No New Symptoms	New Onset Cardiac Symptoms	Relative Risk (95% CI)	P value
	*n* = 1150	*n* = 120		
**Sex *n (%)***				
Male	959 (90.6%)	99 (9.4%)	1.1 (0.7–1.6)	0.80
Female	191 (90.1%)	21 (9.9%)		
**Age (years) *mean (SD)***	25.4 (8.4)	24.6 (7.3)		0.32
**Race *n (%)***				
White	777 (90.7%)	80 (9.3%)	1.0 (0.7–1.5)	0.84
All Other Races	373 (90.3%)	40 (9.7%)		
**Weight (lbs) *mean (SD)***	175.2 (27.6)	176.8 (29.5)		0.55
**Smoking history *n (%)***				
Former/Never	614 (92.7%)	48 (7.3%)	1.6 (1.2–2.3)	0.005
Current/Recent	536 (88.2%)	72 (11.8%)		
**Vaccine Type *n (%)***				
TIV	184 (97.4%)	5 (2.6%)	4.0 (1.7–9.3)	<0.001
SPX	966 (89.4%)	115 (10.6%)		
**SPX Vaccine Type *n (%)***				
ACAM2000	366 (90.4%)	39 (9.6%)	1.2 (0.8–1.7)	0.42
Dryvax	600 (88.8%)	76 (11.2%)		
**SPX Vaccine Dose *n (%)***				
Primary	921 (89.7%)	106 (10.3%)	1.6 (0.9–2.9)	0.17
Secondary	45 (83.3%)	9 (16.7%)		
**Pre-Vaccine Health**				
**Self-Assessment *n (%)***				
Excellent	684 (92.8%)	53 (7.2%)		0.001
Good	435 (87.7%)	61 (12.3%)	1.7 (1.2 to 2.4)	
Fair	30 (83.3%)	6 (16.7%)	2.3 (1.1 to 4.7)	
Poor	0 (0.0%)	0 (0.0%)		
**Physical Limitations *n (%)***	87 (82.9%)	18 (17.1%)	2.0 (1.2–3.0)	0.008

For relative risks, the reference group is listed first.

SD: Standard Deviation.

### Myocarditis/Pericarditis Incidence Post-Immunization

The TIV cohort (n = 189) had no cases of clinical or subclinical MP following immunization (incidence rate 0 per 100,000 (95% CI (0–1952)). In contrast, within the SPX cohort (n = 1081) there were five cases of new onset clinical MP: pericarditis (1), myocarditis (4). All the myocarditis cases were Caucasian males (1 pericarditis case was female), consistent with the demographics of cases previously published [3, 4]. The myocarditis/pericarditis incidence rate for the post-SPX cohort was 463 per 100,000 (95% CI 150–1079 per 100,000). Only two of the five cases sought acute medical care outside of the protocol visits and 3 cases were identified by new onset cardiac symptoms (without seeking acute evaluation) and associated cTnT elevations (peak values 0.05–0.357 ng/ml) with pre-vaccine levels <0.01 ng/ml. As shown in Table 7, when compared to the published background rate of MP in a comparable population of service members not exposed to SPX immunization (detailed in Table 3)[3], the relative risk of clinical MP was 214 fold higher (95% CI (65–558); p<0.001) than the published background rate. It is noteworthy that the epidemiologic retrospective post-SPX rate was 7.5 fold higher than the background rate compared to 214-fold higher rate in this prospective SPX study [3].

**Table 7:** Prospective Cases of New Onset Myocarditis/Pericarditis or cTnT Elevation Following Immunization with Either Smallpox or Trivalent Influenza Vaccine.

Post-Vaccine Event	SPX	Healthy 2002*	TIV	Relative Risk	
	n = 1081	N = 1,390,352	n = 189	(95% CI)	
**Clinical**					
**Myocarditis/Pericarditis** ^‡^	5	30	(0)		
Per 100,000 Incidence Rate	**463**	**2.2**	(0)	**214** ^§^	
95% CI	150–1079	1.9–2.3	0–1950	(65, 558)	
**Possible Subclinical**					
**Myocarditis** ^ǁ^	31		0	
Per 100,000 Incidence Rate	**2868**		**0**	
95% CI	1948–4070		0–1950	(P = 0.016)

*Healthy 2002: DoD Defense Medical Surveillance System pre-SPX MP incidence data.^3^

^‡^ **Prospective clinical myocarditis/pericarditis** cases included 4 Caucasian male cases of probable myocarditis (new onset cardiac symptoms (chest pain, dyspnea on exertion and/or at rest, palpitations) and cTnT elevations ≥0.02 ng/ml with the pre-vaccine level <0.01 ng/ml). The 5^th^ case (female) was acute suspect pericarditis presenting with characteristic chest pain and no cTnT elevations or ECG changes. There were no cases in the TIV prospective study cohort.

^§^ **Comparison of Prospective Smallpox Vaccine Cohort with published historic retrospective epidemiologic estimate of myocarditis/pericarditis disease incidence in comparable population pre-SPX vaccine**: P<0.001.

^ǁ^Subclinical myocarditis is defined by increases in cTnT (above pre-immunization levels) without classic new onset cardiac symptoms. The comparison cohort does not reflect a dynamic change but a single level in time in healthy population subsequently followed for mortality relative risk. **Possible subclinical pericarditis**: There were no cases of possible subclinical pericarditis identified through the blinded ECG series review process.

### Subclinical Myocarditis/Pericarditis Incidence

Possible subclinical myocarditis incidences for SPX and TIV cohorts were based on data from subjects with cTnT measurements at visit 1 (pre-vaccine) and at least one post-vaccine visit showing a dynamic change from baseline that met the case definition. In the absence of new onset cardiac symptoms, 31 (96.8% male) subjects in the SPX cohort demonstrated elevations in cTnT post immunization that met our first criteria for subclinical MP (<0.01 ng/ml pre-immunization with post-vaccine level ≥0.02 ng/ml). These elevated cTnT observations occurred predominantly between days 6 and 13 post-vaccination (median 10 days, range 6–28). In comparison to the SPX cohort, the post-immunization incidence rate of comparable cTnT elevations in the TIV cohort was zero (p = 0.01).

The absolute subclinical cTnT elevations post SPX vaccine ranged from 0.02 to 0.24 ng/ml with the majority (n = 28, 90.3%) ranging from 0.02 to 0.07 ng/ml. All elevations normalized in the 3^rd^ visit if data were available. No subclinical pericarditis cases (based on diagnostic ECG changes post vaccine) were identified. Three subjects had cTnT elevations ≥ 0.10 ng/ml (0.10, 0.10, 0.24 ng/ml) without cardiac symptoms.

The frequency of new elevations of cTnT post-SPX (and meeting our postulated case definition for possible subclinical myocarditis) was significantly greater than in the post-TIV group (p = 0.016) as detailed in Table 7. Relative risk could not be calculated because there were no occurrences in the TIV immunized group.

The majority of MP cases (except one case of pericarditis) and possible subclinical MP were Caucasian males receiving the vaccine for the first time. All MP and subclinical MP signs and symptoms resolved spontaneously with conservative management.

## Discussion

This manuscript describes the first prospective post-licensure study, using surveillance time points correlating with peak windows of post-vaccine systemic clinical symptoms and increases in immune activation/inflammation biomarkers [17], to define the incidence of both clinical MP and possible subclinical (without cardiac symptoms) cardiac injury following SPX immunization. Utilizing active surveillance, we identified a significantly higher incidence (463 per 100,000) of clinical MP (compared to previously published background rates in a comparable population). Prior to the present study, the incidence of MP following smallpox vaccination was estimated from passive surveillance registries and population databases, with an inherent bias towards underestimation of disease incidence. For example, MP or cardiac adverse events were not identified in the smallpox vaccination experience of the Israeli Defense Force or in a review of the U.S. experience with SPX immunization in the 1970s [18–19]. However, older studies from northern European countries suggested a link between smallpox vaccine and MP [20–22]. The estimates of incidence rates for MP were significantly underestimated in early reports on the SPX program [23–24].

Our study is the first to prospectively define the incidence of new onset cardiac symptoms in temporal association with SPX and TIV immunization. Despite no significant differences in pre-vaccine health self-assessment between the cohorts, and fewer reported physical limitations in the SPX cohort, there was a significantly higher incidence (4-fold) of new onset cardiac symptom(s) post-SPX (10.6%) compared to post-TIV (2.6%). This difference suggests a unique association with vaccinia immunization. The peak immune inflammatory activation pattern following SPX vaccine occurs between day 8–9 (with a range of day 4–27) post-SPX and includes a predominantly Th1 cytokine pattern (interferon-γ, tumor necrosis factor-α, interferon-inducible protein-10, interleukin-6, granulocyte/granulocyte-macrophage-colony-stimulating-factor, etc.) [17]. These cytokine elevations mirrored our observed timeline for peak incidence of cTnT elevations, new onset cardiac symptoms and cases of MP, suggesting inflammation as the mechanistic link to the elevated troponins as well as symptoms. Other physical symptoms following SPX have been correlated to various cytokine elevation patterns [17, 25–26]. While no studies have as yet identified genetic risk biomarkers for the development of myocarditis, cardiac symptoms or troponin release injury post immunization, other adverse events or systemic symptoms have been linked to genetic polymorphism in the cytokine gene for IL4 [26].

The strengths of this study included baseline comparisons for each subject’s post-vaccine measures and a robust analysis that controlled for other baseline differences between vaccinated groups. The consistency among SPX and TIV cohort participants with an occupationally related physical fitness requirement ensured a reasonable expectation of pre-vaccine cardiovascular health. Additionally, the independent review of MP cases by cardiologists experienced in myocarditis, blinded ECG interpretations, and serial clinical evaluations minimized any potential for confirmation bias.

One explanation for the potential underestimation of the true incidence of MP in healthy, physically active populations (with a low risk of coronary artery disease), regardless of cause, is the clinical bias that chest pain would more likely be interpreted as musculoskeletal (e.g., myalgias) or part of “body aches.” For example, symptom diary data from 1000 civilian laboratory workers receiving SPX did not mention chest pain [23]. Because of the infrequent use of endomyocardial biopsy, the true incidence of histologically confirmed myocarditis in the general population is not known [24]. Evolving understanding of clinically significant troponin levels may explain prior lower rates of MP clinical diagnosis [27–29].

Unlike Ahlborg’s earlier study in military recruits [20], our study did not identify any subclinical cases of pericarditis through serial ECGs. Given the population of healthy active subjects enrolled, there were a high number of abnormal readings that were ultimately identified as normal variants due to early repolarization. The fact that the ECG serial monitoring did not identify additional potential “subclinical cases” is consistent with the published report by Sano et al. [30].

The use of dynamic cTnT changes before and after immunization as a surrogate biomarker for a case definition of possible subclinical myocarditis has not been used previously. A review of the next generation modified vaccinia Ankara vaccine studies measured pre-post changes at the 14 day time-point but did not develop such a standardized case definition nor did it address using the subject as their own control to define dynamic changes, focusing on clinical case detection alone [31]. These studies also did not focus on timing of measurements in parallel to potential peak immune responses when inflammatory stressors might trigger a cardiac reaction. While assumptions about benign troponin release from the myocardium have been made, there is a growing body of literature suggesting that even in generally healthy populations with no known cardiac disease risk, small elevations in troponin (stratified below the levels measured by the assay in this study) are associated with increased risk of all cause and cardiovascular mortality [14, 15, 32–36]. Therefore, the rate of reported elevations in this study may actually be an underestimate of the true incidence of vaccine related subclinical myocarditis.

The Global Task Force Guidelines for the Application of the Universal Definition of Myocardial Infarction defines the diagnostic troponin level for acute cardiac injury as ≥99^th^ percentile of the upper-reference-limit which, for the cTnT assay used in this study, is <0.01 ng/ml [33]. Recent publications indicate there is clinical significance for levels below this value (in the less than 10 picogram/ml ranges) when higher sensitivity assays are used [12, 34–36]. There is no defined threshold for the diagnosis of myocarditis. Reports with myocardial biopsy data suggest lower cTnT thresholds in order to optimize diagnostic sensitivity for acute myocarditis [28]. In a report by Lauer et al, 44% of patients with cTnT levels below 0.10 ng/ml had evidence (histologic or immunohistologic or both) of myocarditis on endomyocardial biopsy, and cTnT levels for all patients with histologic evidence of myocarditis suggested a clustering of cases between undetectable and 0.10 ng/ml [29]. None of these studies involved cohorts that were asymptomatic.

This is the first report providing a case definition for possible subclinical myocarditis along with an estimate of the incidence rate in a SPX immunized cohort. However, the specificity as well as sensitivity of the proposed case definition remains to be validated with open questions about the long-term prognosis or pathogenesis. While the disease confounders usually associated with troponin elevations [15] are not known to be present in the study populations, the cause for the troponin release could be attributed to other causes. However, Wallace et al. [12,15] showed that in an older population than ours, free of diabetes mellitus, hypertension, chronic kidney disease, left ventricular hypertrophy, congestive heart failure, history of myocardial infarction, low ejection fraction or BMI>30 (n = 1060), no subjects were observed with detectable cTnT (>0.01 ng/ml). The fact that there were no changes in the TIV group supports that whatever the mechanism, it remains probable that the transient troponin elevations are linked to the immune activating stressors associated with the SPX vaccination. Cardiac myosin molecule is a dominant auto-antigen in animal models of myocarditis, presumably after release from the injured heart, and raises additional questions about the long-term risks of cardiac troponin release [37]. Inflammatory cytokine pathways, activated by SPX immunization, have been associated with cardiac inflammation [38, 39]. Given the patchy nature of myocarditis and the benign clinical course in the post-vaccine MP cases, cardiac biopsies would not necessarily have been helpful and would not be clinically indicated (benefit-risk for such an invasive procedure could not ethically be justified).

A limitation of the study is the small size of the TIV cohort relative to the SPX cohort, which limited our ability to precisely estimate the adjusted relative risk of clinical and possible subclinical MP following influenza immunization. However, based on the limited published case reports of MP following TIV [40, 41], the incidence must be very rare and not clearly defined as increased compared to the background rate of disease presentation. Again, a balancing strength for the smaller TIV cohort size is the use of each subject as their own control.

The dynamic change in cTnT levels post SPX vaccine may reflect underlying subclinical cardiovascular disease as well as myocarditis given the growing evidence that atherosclerotic/ischemic cardiovascular disease progression also involves immune inflammatory mechanisms [39]. This study raises the question whether or not inflammation in response to primary SPX immunization or other conditions that stimulate immune activation represent a new “stress test” that unmasks ischemic disease versus non-ischemic inflammatory myocarditis. The population studied was a healthy and physically active group of young men and women and would be expected to have a low risk of ischemic cardiovascular disease. It is noteworthy that Eckart et al. did not show any epidemiologic evidence of increased acute atherosclerotic cardiovascular events in the 30 day period following SPX immunization in a comparable study population [42]. In contrast, Jacobson et al. showed that there is an increased risk for hospitalization post-smallpox vaccine with the highest hazard ratios associated with coding combinations for inflammatory (non-atherosclerotic) cardiac disease [43]. The long term risk of cardiovascular disease with transient elevations of cTnT in our study population remains to be defined.

The confounder of exercise related changes in cTnT measurements is an unlikely limitation. cTnT levels may be elevated after intense exercise but controversy exists as to the long-range clinical implications and, whether these are benign physiologic changes or a marker of poor prognosis [44–49]. This study was not designed to specifically quantify exercise in temporal association with the visits and this may be considered another limitation for accurately defining the significance of cTnT elevations post-SPX. The fact that no changes were seen in a healthy, active TIV immunized population further supports that there is a specific effect in SPX-vaccinees that causes transient cardiac muscle injury. High-sensitivity cTnT studies of pre-post exercise stress testing in patients with potential cardiovascular disease only show small increases in cTnT (in the less than 10 picogram range per ml) and this biomarker was associated with future prognostic risk. Picogram range elevations would not have been detected in the assay used for this study [36, 49–51].

It is noteworthy that the publication by Elizaga et al. [52], reporting on cardiac adverse events in healthy subjects receiving modified vaccinia Ankara vaccines, does not address the type of troponin I assay used or definition for elevation. In addition, the timing of post-vaccine testing was beyond the window of peak detection identified in our study. As troponin assay technology and prognostic significance continue to evolve, there is a need for standardized guidelines for their use within drug and vaccine safety surveillance studies. Defining long-term cardiac morbidity and mortality risk resulting from adverse drug reactions remains a significant challenge for post-licensure safety surveillance.

Our study identified a rate of myocarditis/pericarditis following SPX immunization that is significantly higher than previously described, and highlights the challenges of post-licensure vaccine safety surveillance to identify adverse events that are not well understood or previously unrecognized. Applying the incidence described in this study to a SPX immunization program that has delivered over 2 million doses, largely to young, healthy primary vaccinees, there are potentially thousands of vaccine associated cases, many undiagnosed because of self-medication or lack of provider awareness [53]. In our study, 3 of the 5 clinical cases would not have sought medical care without study interventions including enhanced education and surveillance. The recognition of potential adverse events following immunization requires accurate diagnosis of new onset clinical symptoms. Our study reinforces the need, as part of all post-vaccine (and potentially new drug related) adverse events surveillance, to include specific standardized inquiry about new onset cardiac symptoms and to highlight the value of dynamic pre to post immunization cardiac troponin increases as a potential biomarker of risk in future safety surveillance studies.
